# Durability of Fibre-Reinforced Calcium Aluminate Cement (CAC)–Ground Granulated Blast Furnace Slag (GGBFS) Blended Mortar after Sulfuric Acid Attack

**DOI:** 10.3390/ma13173822

**Published:** 2020-08-29

**Authors:** Wei Fan, Yan Zhuge, Xing Ma, Christopher W. K. Chow, Nima Gorjian, Jeong-A Oh, Weiwei Duan

**Affiliations:** 1 UniSA STEM, University of South Australia, Adelaide 5095, Australia; wei.fan@mymail.unisa.edu.au (W.F.); Xing.Ma@unisa.edu.au (X.M.); Christopher.Chow@unisa.edu.au (C.W.K.C.); jeong-a.oh@mymail.unisa.edu.au (J.-A.O.); Weiwei.Duan@unisa.edu.au (W.D.); 2 South Australian Water Corporation, Adelaide 5000, Australia; Nima.GorjianJolfaei@sawater.com.au

**Keywords:** calcium aluminate cement (CAC), ground granulated blast furnace slag (GGBFS), strain hardening behaviour, engineered cementitious composites (ECC), sulfuric acid attack

## Abstract

Concrete wastewater infrastructures are important to modern society but are susceptible to sulfuric acid attack when exposed to an aggressive environment. Fibre-reinforced mortar has been adopted as a promising coating and lining material for degraded reinforced concrete structures due to its unique crack control and excellent anti-corrosion ability. This paper aims to evaluate the performance of polyethylene (PE) fibre-reinforced calcium aluminate cement (CAC)–ground granulated blast furnace slag (GGBFS) blended strain-hardening mortar after sulfuric acid immersion, which represented the aggressive sewer environment. Specimens were exposed to 3% sulfuric acid solution for up to 112 days. Visual, physical and mechanical performance such as water absorption ability, sorptivity, compressive and direct tensile strength were evaluated before and after sulfuric acid attack. In addition, micro-structure changes to the samples after sulfuric acid attack were also assessed by X-Ray Diffraction (XRD) and Scanning Electron Microscopy (SEM) to further understand the deterioration mechanism. The results show that overall fibre-reinforced calcium aluminate cement (CAC)-based samples performed significantly better than fibre-reinforced ordinary Portland cement (OPC)-based samples as well as mortar samples in sulfuric acid solution in regard to visual observations, penetration depth, direct tensile strength and compressive reduction. Gypsum generation in the cementitious matrix of both CAC and OPC-based systems was the main reason behind the deterioration mechanism after acid attack exposure. Moreover, laboratory sulfuric acid testing has been proven for successfully screening the cementitious material against an acidic environment. This method can be considered to design the service life of concrete wastewater pipes.

## 1. Introduction

Sewerage networks and wastewater systems are significant urban infrastructures to modern societies worldwide, conveying the safe transport of sewage from households and industrial sites to wastewater treatment plants. However, these valuable assets often suffer serious corrosion and deterioration issues due to external aggressive acid attack, resulting in a huge loss to the economy annually. Sulfuric acid (H_2_SO_4_), generated by sulfur/sulfide-oxidising bacteria, has been recognised as the primary acid in wastewater environment. Normal concrete pipes made of ordinary Portland cement (OPC) have minimal resistance to sulfuric acid attacks, as this substance unable to resist any solution lower than pH of 3. The effect of sulfuric acid (H_2_SO_4_) on concrete is complex as it is a combination of acid and sulfate attack. The chemical reaction of sulfuric acid attack that takes place in concrete sewerage can be seen as follows [1]:

Acid attack: Ca(OH)_2_ + H_2_SO_4_ → CaSO_4_·2H_2_O (gypsum) (1)

Acid attack: 3CaO·2SiO_2_·3H_2_O + H_2_SO_4_ → CaSO_4_·2H_2_O + Si(OH)_4_ (2)

Sulfate attack: 3CaSO_4_ + 3CaO·Al_2_O_3_·6H_2_O + 25H_2_O → 3CaO·Al_2_O_3_·3CaSO_4_·32H_2_O (3)

Gypsum formation occurred in Equations (1) and (2), whereas ettringite is formed from Equation (3). It is known that a solid volume expansion caused by ettringite formation leads to internal pressure increase and eventually cracking the concrete. The deterioration rate can be escalated especially in sewerage pipes as the corroded layers can be removed by the sewerage flow. The total value of these network assets is estimated to be about one trillion dollars in the USA and 100 billion dollars in Australia [2]. As the aging of sewer infrastructure continues, it is challenging to maintain the aged and degraded concrete sewerage pipes for municipalities. The conventional open-cut replacement method for underground sewerage pipes is very costly and time-consuming. Thus, developing an effective repair material to extend the service life of concrete sewerage pipe is urgently required.

Strain hardening cementitious composite (SHCC) has been increasingly adopted for the repair of aged concrete structures when subjected to adverse environmental impact. Liu et al. [3] found out that strain hardening cementitious composite (SHCC), exposed to sulfate and sulfate-chloride solution after 200 days, can still exhibit significant multiple cracking behaviour by achieving a tensile strength of 6.29 MPa and a tensile strain capacity of 2.14%, while the compressive strength of conventional mortar samples decreased by up to 35.7% after 420 days of being exposed to sulfate and sulfate-chloride solution. The dual effect of freeze-thaw cycles and NaSO_4_ solution (5.0% (wt%)) were investigated by Özbay et al. [4] on SHCC. The results show that the ductility of SHCC was remarkably decreased after 300 freeze–thaw cycles. The self-healing capacity of SHCC was also reported by Liu et al. [5], in that the sample can autogenously heal after sulfate exposure and tends to heal more completely and faster in NaSO_4_ solution than in water. In addition, due to SHCC’s tiny cracking width, the permeability of liquid could be maintained at a lower level even in the cracking stage; therefore, several hydraulic infrastructures, including water irrigation channels and dams, have been repaired or retrofitted by SHCC, prolonging the service life. Mitaka-dam (Hiroshima prefecture, Japan) suffered severe water abrasion that led to water leakage. An SHCC layer was sprayed onto the upstream side of the dam wall for protection and later performed extraordinarily in resisting water penetration [6]. Spray application of SHCC was applied on the Seridanno Channel in Toyama Prefecture and trowel application was used on the Central Main Channel in Shiga Prefecture and in Japan in 2005—the performance of freezing and thawing, carbonation resistance and cracking resistance of the irrigation channels were improved [7]. SHCC lining was proven to be as an effective approach for Coppermills Water Treatment Facility retrofitting in North London, UK. The combine advantages of water tightness, durability and tensile capacity makes SHCC fibre-reinforced mortar able to withstand tension and simultaneously keeping a crack width below 50 μm. Corrosion of steel reinforcement was avoided after the repair [8]. Given the benefits of chemical resistance and water tightness, fibre-reinforced mortar can potentially be used for wastewater infrastructure rehabilitation. However, currently there is no published literature reporting the durability of strain hardening cementitious composite exposed to sulfuric acid-induced sewer corrosion.

To better utilise SHCC for the sewerage environment, it requires the modification of its cementitious binder, as normal ordinary Portland cement is vulnerable to acid attack. Thus, replacement of OPC with corrosion-resistant cement for sewerage pipe repair is necessary. Calcium aluminate cement (CAC)-based concrete can perform six times more effective acid-resistance than OPC-based concretes [9]. In OPC-based binder systems, the main hydrates are CH and C-S-H, while due to the potential antimicrobial properties of Al, CAC-based binder systems exhibit superior performance ascribed to aluminium hydroxide (Al(OH)_3_) and the formation of a pore filling Al(OH)x gel acted as a buffer under acid attack, which shows great cohesion of the degraded layer [10]. A longer chain of dissolution and neutralisation was provided by various CAC hydrates (CAH_10_, C_2_AH_8_, C_3_AH_6_, and AH_3_). AH_3_ is recognised as a stable compound, and its pH value is 3–4, which has great potential in neutralising acid attacking at such pH values. This is the main reason that CAC-blended binder usually has a higher acid neutralisation capacity than OPC-based binder systems [11]. The overall neutralisation reaction is as follows [12]:

C_3_AH_6_ + 2AH_3_ + 24H^+^ → 3Ca^2+^ + 6Al^3+^ + 24H_2_O (4)

AH_3_ + 6H^+^ → 2Al^3+^ + 6H_2_O (5)

Ground granulated blast furnace slag (GGBFS) has been well recognised as an effective supplementary cementitious material (SCM) to increase the acid resistance of reinforced concrete structures. The secondary hydration reaction of GGBFS can inhibit the amount of Ca(OH)_2_ and produce additional calcium-silicate-hydrate gel, which refines the pore structures of concrete and thereby increase the acid resistance [13]. Apart from the chemical resistance, incorporation of GGBFS can also solve the conversion issues of pure CAC systems [14].

In this paper, the authors take a first step to investigate the sulfuric acid resistance of strain hardening PE fibre-reinforced CAC-GGBFS blended mortar. The work aims to elucidate a full understanding of the durability performance of the fibre-reinforced mortar under an aggressive environment that represents the sewerage conditions. The influence of sulfuric acid attack on physical and mechanical properties including physical properties, compressive strength and tensile strain hardening behaviour was studied. In addition, microstructure analysis was also conducted to gain an insight into the physical mechanism underlying the macrostructural observation.

## 2. Experimental Program

### 2.1. Materials

**Figure 1:**
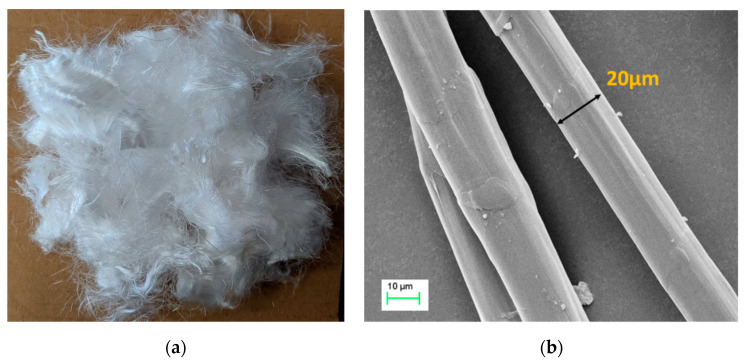
Photo (**a**) and SEM image (**b**) of polyethylene (PE) fibre.

Various short-cut fibres were mixed in the cementitious matrix to increase its toughness, bending, durability and modify the failure mode. The mechanism of fibre reinforcing prevents the growth and generation of macroscopic and microscopic cracks via the fibre bridging effect. Due to the hydrophobic nature with a simple chemical structure, polyethylene (PE) fibre, showing good tensile strength and better chemical resistance, was adopted in this study. An SEM image of the PE fibre surfaces texture is shown in Figure 1. The 18 mm monofilament PE fibre has a smooth surface and circular cross-section. The mechanical and geometric properties of PE fibre are shown in Table 1.

The ingredients of the mix design included CAC, OPC, GGBFS, fine silica sand as well as PE fibre. The slag to cement ratio was chosen at a mass proportion of 1.5 and 3.8, and PE fibres were employed with a volume fraction of 1% [15,16]. OPC-based fibre-reinforced mortar and CAC-based plain mortar were also prepared as a reference with the slag to cement ratio of 1.5.

**Figure 2:**
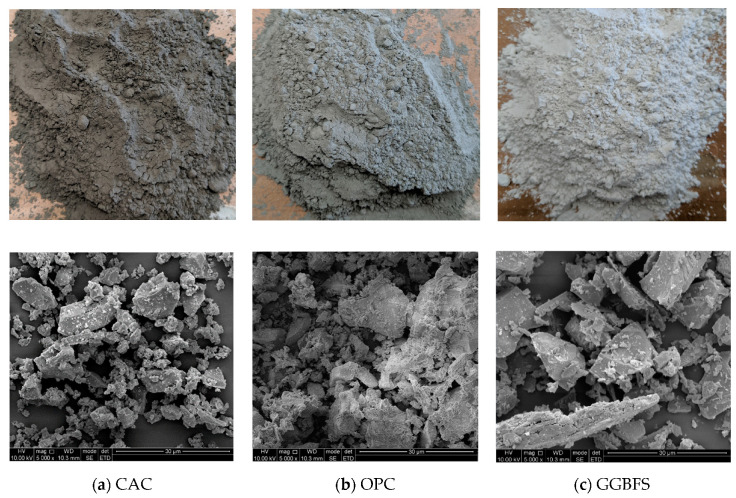
Photo (**above**) and SEM image (**below**) of cementitious material.

The mix design and mechanical properties were discussed in detail in the authors’ previous work [17]. The four mix designs were named as CAC-GGBFS 1.5, CAC-GGBFS 1.5PE, CAC-GGBFS3.8PE and OPC-GGBFS 1.5PE, with an average flow diameter of the four mixtures tested as 340, 310, 300 and 390 mm, respectively. OPC-GGBFS 1.5PE has the highest compressive strength of 74.73 MPa at 28 d, while CAC-GGBFS1.5PE only has a compressive strength of 59.76 MPa. With the increased s/c ratio, a strength reduction is observed in CAC-GGBFS 3.8PE of 25.54 MPa. Plain mortar sample CAC-GGBFS 1.5 has a compressive strength of 49.26 MPa. Detailed specifications of the mixing ingredients are given in the authors’ previous work [17]. Photos and SEM images of the raw cementitious material used are shown in Figure 2. The optimum mix proportions are shown in Table 2.

### 2.2. Sample Preparation and Testing Procedure

**Figure 3:**
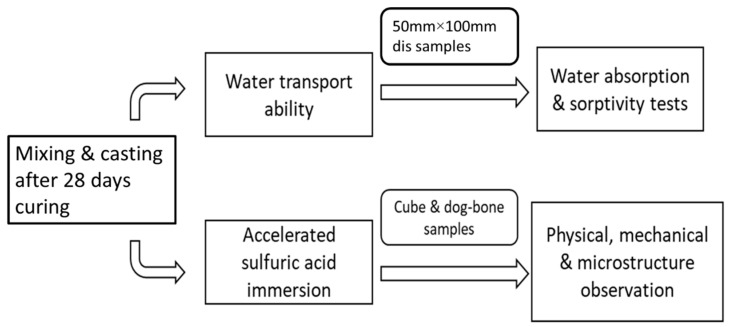
Testing procedure.

All PE fibre-reinforced CAC-GGBFS blended mortar mixtures were prepared according to the author’s previous work [17] to study the mechanical properties. All mixtures were cast into disk, cube and dog-bone shape. All samples were kept in the moulds for up to 24 h at ambient temperature, and later they were demoulded and placed to curing room for 28 days. After 28 days of curing, specimens were divided into two parts for durability test. First, 50 mm × 100 mm disk samples were prepared for the water transport properties test, then cube and dog-bone shape samples were used for the accelerated sulfuric acid attack test. The overall test procedure is shown in Figure 3.

### 2.3. Water Transport Properties

After 28 days of curing, the water absorption as well as initial capillary sorptivity of the four mix designs were measured. This value can provide the significance of rate and tendency where liquid (e.g., water or acid) was transmitted and absorbed into the samples because of capillary force. This capillary behaviour can have great impact on the degradation behaviour of the tested specimens.

#### 2.3.1. Absorption and Voids

Three samples from each mix were taken to test the water absorption after 28 days of curing in accordance to ASTM C-642 [18]. Firstly, samples were dried in the oven at a temperature of 100–110 °C for 24 h, following by cooling at room temperature, then specimen mass was measured. The samples were saturated in water for two days at about 21 °C, and the weight was measured after drying their surfaces until the mass difference was within 0.5% of the last two measurements. Water absorption was calculated with the ratio of mass of the water adsorbed in the saturated sample to the mass of the dried sample. The water absorption of the samples was then determined using Equations (6) and (7):

(6) $$Absorption=\frac{W_{SD}-W_{dry}}{W_{dry}}\times 100$$

To determine the volume of pores, the hydrostatic weight (*W_HD_*) of the specimens was also determined and the volume of pores was calculated as follows:

(7) $$Void\;content\;\left(\%\right)=\frac{W_{SD}-W_{dry}}{W_{dry}-W_{HD}}\times 100$$

where *W_dry_* is the mass of the oven-dried sample (g) and *W_SD_* is the surface dried mass of the sample after immersion (g).

#### 2.3.2. Sorptivity

**Figure 4:**
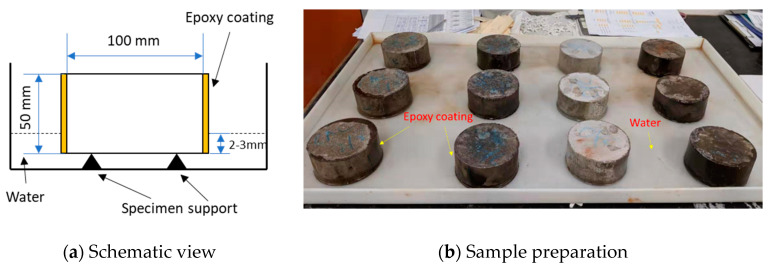
(**a**,**b**). Test set-up for sorptivity test.

Sorptivity is known as the penetration of liquid into porous matrix under unsaturated conditions associated with the capillary suction. The specimen weight gain was measured in a set timeframe to determine the sorptivity. This measurement can give an indirect index on the porous structure in the near surface zone. Samples were prepared with a 100 mm-diameter, 50 mm-thick disc, cut from the 200 mm-long 100 mm-diameter cylinders with a diamond saw at 28 days. Then the specimens were placed into an oven and dried at 50 ± 5 °C until the difference in mass between any two measured values is less than 0.5% of the lowest value obtained. One surface of the specimen only could be in contact with water at the depth between 2 and 3 mm and the side surface of each specimen was coated with an epoxy to make sure the flow travelled through only one direction to the specimen, which is shown in Figure 4. Absorption rate (mm^3^/mm^2^) was plotted versus the square root of time (min^1/2^). The absorption of water into concrete can be described by the square root of time relationship according to ASTM C1585 [19]. The absorption rate was calculated with the mass (g) change divided by the cross-sectional area of the disc sample (mm^2^) and the water density at the recorded temperature (g/mm^3^). The slop of the resulting curve defines the sorptivity of the specimen during the initial 6 h of testing.

### 2.4. Accelerated Sulfuric Acid Immersion

**Figure 5:**
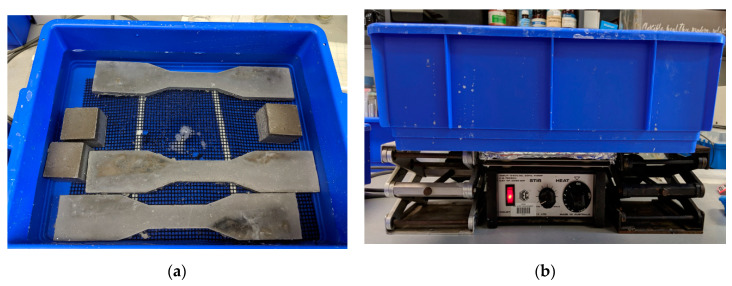
(**a**,**b**)**.** Sulfuric acid bath with magnetic stirrer.

While in situ biogenic tests can take up to several years and a biogenic chamber is largely infeasible in most civil engineering labs, as an alternative, the exposure of PE fibre-reinforced CAC-GGBFS blended mortar to chemical sulfuric acid was adopted to study the effect of this type of material under the sewerage environment. The test is based on Gu et al.’s [20] and Sturm et al.’s [21] method. For each group of mixtures, three 50 × 50 × 50 mm cubes for compressive tests and three dog-bone specimens for uniaxial tensile tests were prepared for sulfuric acid immersion, as shown in Figure 5a. Specimens were demoulded after 24 h, then they were stored in a curing room with a temperature of 23 ± 3 °C until the age of 28 days prior to being exposed to sulfuric acid environments. Prior to sulfuric acid immersion, all samples were immersed in tap water at 23 °C for 2 days to saturate the pores. Then, the samples were transferred to a 3% (*w/w%*) sulfuric acid bath with a pH value of 0.55. The specimens in the sulfuric acid bath were immersed at least 10 mm below the surface in a volume of 6 L of the acid solution. The samples were placed on plastic grids and the sulfuric acid constantly stirred by a magnetic stirrer to ensure the samples were homogeneously exposure to acid as shown in Figure 5b. To reduce the impact of evaporation, lids were covered on the test tanks. The pH levels of the sulfuric acid solutions were monitored using a portable digital pH meter. To maintain a relatively stable pH, the sulfuric acid was renewed weekly.

Visual observations were performed as physical assessments of the specimens which were taken from acid container after 0, 7, 28, 60 and 112 days of exposure. The assessment in visual observation involved investigating the crazing, cracking and change in colour on the surface after different exposure days. Then, cross-sections of cubes from each exposure time were sawed and analysed on regions located close to the exposed surfaces to discover corrosion layer growth. Further, X-ray diffraction (XRD) analysis was conducted on the samples taken from 0 and 112 d exposure surface to evaluate the reaction products after deterioration. XRD analysis were performed to analyse the mineralogical phases in powdered samples. These powders were operating at 40 KV and 150 mA with Cu–Ka radiation with a wavelength of 0.154056 nm, in the 2θ range of 5–50° and at the scan rate of 6° (2θ) per minute. The phase identification and analysis of the scan patterns were conducted using the computer software 204 Diffrac Eva (Version 4.1, manufacturer, city, country) and the ICDD PDF-4+2018 database. The scanning electron microscopy (SEM) observation was conducted on small pieces from the fractured section of the dog-bone specimens after the uniaxial tension test to study the surface morphology of the PE fibre and to find out the failure modes after sulfuric acid exposure.

Mechanical performance was evaluated based on compressive strength and tensile stress-strain relationship. Three cubic 50 mm specimens were tested at different ages (0, 7, 28, 60 up to 112 days) after 3% (w/w%) sulfuric acid solution immersion to determine the compressive strength as a function of time. After sulfuric acid immersion, the specimens were dried at room temperature for 24 h and then the compressive and direct tensile behaviour was tested. Compressive tests on cube specimens were carried out at a loading rate of 1300 ± 300 N/s in accordance with AS1012-14 [22]. Three dog-bone specimens were adopted to conduct uniaxial tensile tests according to the Japan Society of Civil Engineers (JSCE) recommendations for direct tension testing of high-performance fibre-reinforced cementitious composites [23]. Uniaxial tensile loading was applied on the dog-bone specimens under displacement control at a rate of 0.5 mm/min. Two external linear variable differential transducers (LVDTs), with a gage length of approximately 80 mm, were attached on each side of the specimen to measure the tensile strain. The detail setup of compressive and direct tensile tests can be found in [17].

## 3. Results and Discussion

### 3.1. Water Transport Properties

The durability of cementitious material is directly influenced by water transport into the microstructure as the porous nature of cementitious material can let the deleterious material penetrate through interconnected pores. The micro-structure of cementitious material can impact on its durability; thus, the results of water transport properties will affect the sulfuric acid immersion test.

#### 3.1.1. Void Content and Water Absorption

**Figure 6:**
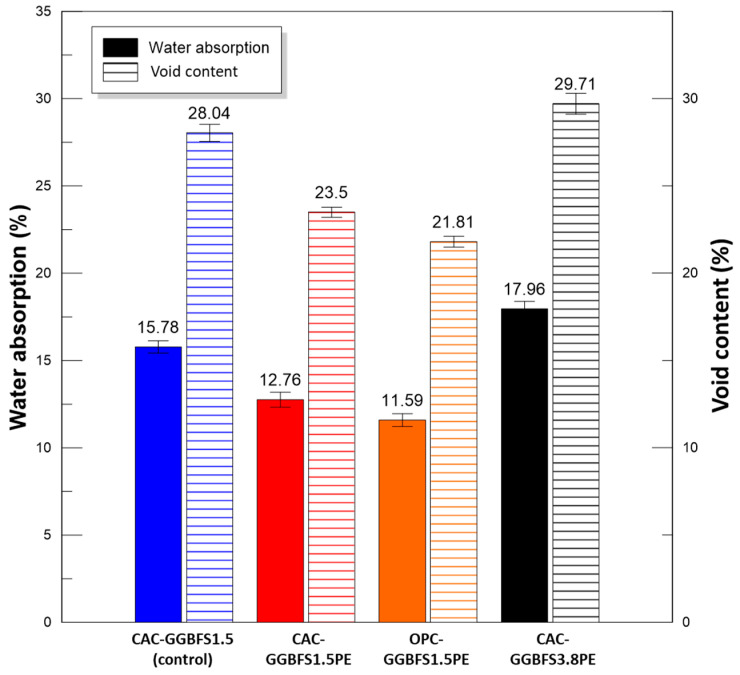
Water absorption and void content (average values).

The average values of void content and water absorption determined after 28 days of curing are shown in Figure 6. Incorporated with PE fibres, the CAC-GGBFS1.5PE sample showed a lower water absorption and lower porosity to the corresponding CAC-GGBFS1.5 samples without fibres. When the slag to cement ratio increased from 1.5 to 3.8, both water absorption and void content increased, which may be attributed to the higher content of GGBFS, resulting in a slow pozzolanic reaction due to an insufficient curing period. OPC-based samples showed the lowest value of water absorption and void content of 11.59% and 21.81%, respectively, which means that OPC hydrates have a denser microstructure than CAC hydrates under normal environmental conditions.

#### 3.1.2. Sorptivity

**Figure 7:**
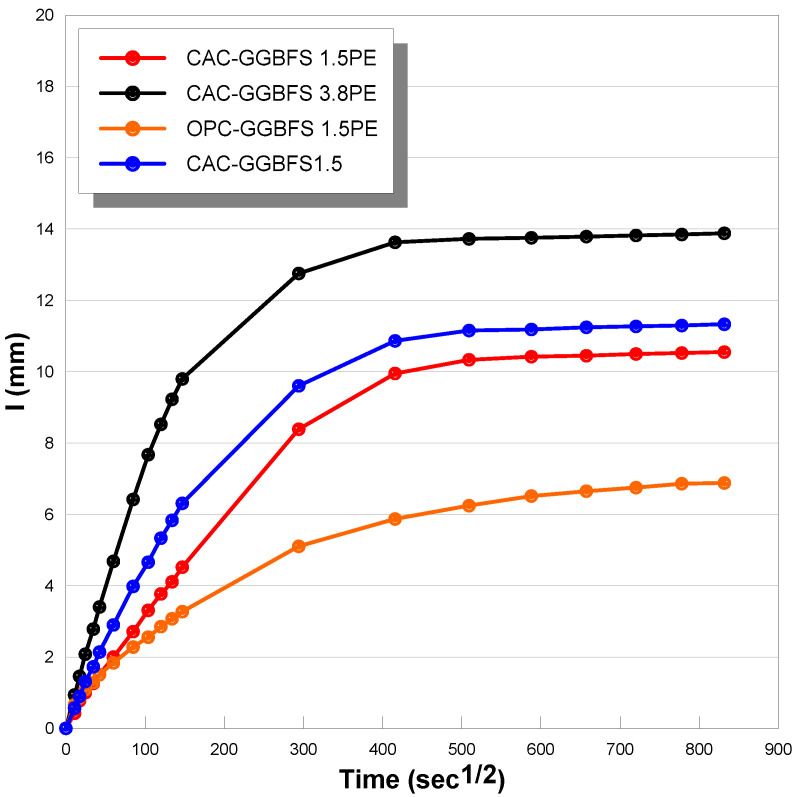
Mass gain verse square root of time (sorptivity test).

Sorptivity is related closely to the capillary capacity and the tendency of concrete material absorbing liquids through capillary pressures. Water acts as the acid carrier when transporting into porous media, thereby sorptivity can provide significant information regarding the degradation rate from acid attack. Cumulative water absorption (normalized per unit surface area) versus the square root of time is plotted in Figure 7. The cumulative volume of water absorbed per unit surface area (mm^3^/mm^2^) in all the mixtures increased with the square root of time. As with water absorption under submerged conditions, the sorptivity coefficient of CAC-GGBFS1.5PE is slightly lower than CAC-GGBFS1.5 after adding PE fibres. As the slag to cement ratio increased from 1.5 to 3.8, the initial and secondary sorptivity coefficients of CAC-GGBFS3.8PE increased by 30% due to the increased void contents. OPC-GGBFS1.5PE still showed the lowest sorptivity coefficient both at the initial stage and secondary stage, which means that under normal circumstances, replacement of OPC with CAC did not inhibit the ingress of aggressive agents into the concrete.

### 3.2. Physical and Mechanical Properties Assessment after H_2_SO_4_ Exposure

#### 3.2.1. Visual Appearance

The visual assessment was performed on each mixture to observe any crazing, cracking and change in colour. Table 3 listed the visual observations of CAC-GGBFS1.5, CAC-GGBFS1.5PE, CAC-GGBFS3.8PE and OPC-GGBFS1.5PE, from 0 to 112 days of exposure in a 3% (w/w%) sulfuric acid environment. From 0 to 7 days, it can be seen that CAC-based cubic samples have an initial smooth greyish surface, while OPC-based cubic samples have a white surface colour only 7 days after sulfuric acid exposure. The products are believed to be gypsum, this is due to the degradation of C-S-H in the OPC-based sample dissolved in sulfuric acid. From 28 to 60 days, CAC-based samples remained a greyish colour, whereas OPC-based samples already showed crazing on the edge. After 112 days of exposure to the sulfuric acid solution, the CAC-GGBFS1.5 cubic sample was cracked with surface spalling off, ascribed to the expansion product gypsum which caused an increase in solid volume and leads to an increase in internal pressure [24]. However, due to the fibre bridging effect, CAC-GGBFS1.5PE and CAC-GGBFS3.5PE samples maintained their structure integrity with a mild degree of corrosion on the surface. Due to the rapidly dissolved calcium hydroxide, the OPC-based system opened the porosity to a deeper penetration of the sulfuric acid and shown a severe corrosion with a thick and soft surface at the end of sulfuric acid exposure. From the visual comparison, in addition to the development of crazing and cracking, the OPC-based and plain mortar without fibre samples obviously showed a significant deterioration and loss of material on the surface.

#### 3.2.2. Change of Cross-Section

In addition to the surface observation, each cubic sample was cut in half with a diamond saw to investigate the thickness of the gypsum layer growth. Table 4 presents the change in cross-sectional area of each mixture group after 0, 7, 28, 60, and 112 days of sulfuric acid exposure. The area change is indicated by the growth of the white gypsum layer on the surface of each specimen. Layer thickness was measured by a Vernier scale at four sides, then the average value was calculated. It can be seen from Table 4 that the OPC-based sample had the most severe cross-section change, with an average thickness of 5.095 mm after 112 days of immersion, corresponding to the surface visual appearance. The gypsum layer thickness grew almost 5 mm in 112 days. While the two CAC-based mixtures showed only 3 and 2 mm gypsum thickness development from 7 days, similar results were also found in [25]. Without fibre addition, CAC-GGBFS1.5 showed cracks inside the sample.

It is interesting to note that although OPC-GGBFS1.5PE showed the lowest water absorption and sorptivity coefficient at 28 days, which indicates a better physical property of resisting water penetration, it performed worst under acid attacking compared to CAC samples. In contrast, the CAC groups had a better performance under an aggressive environment due to its different minerology. A more in-depth analysis was conducted in the following section to obtain a thorough understanding of the corrosion mechanism.

#### 3.2.3. XRD Analysis on Mortar Surface

**Figure 8:**
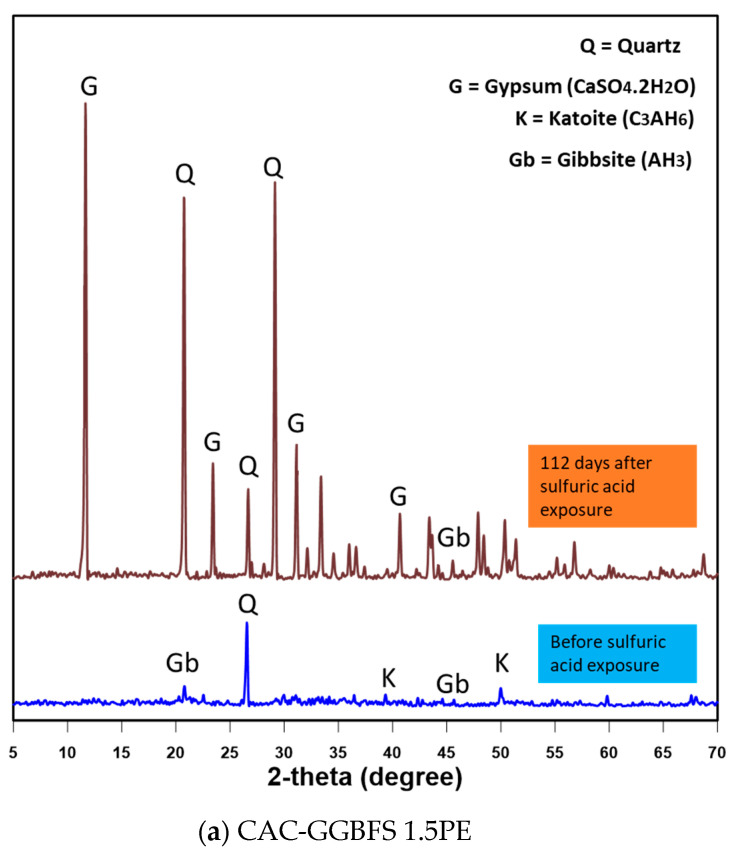

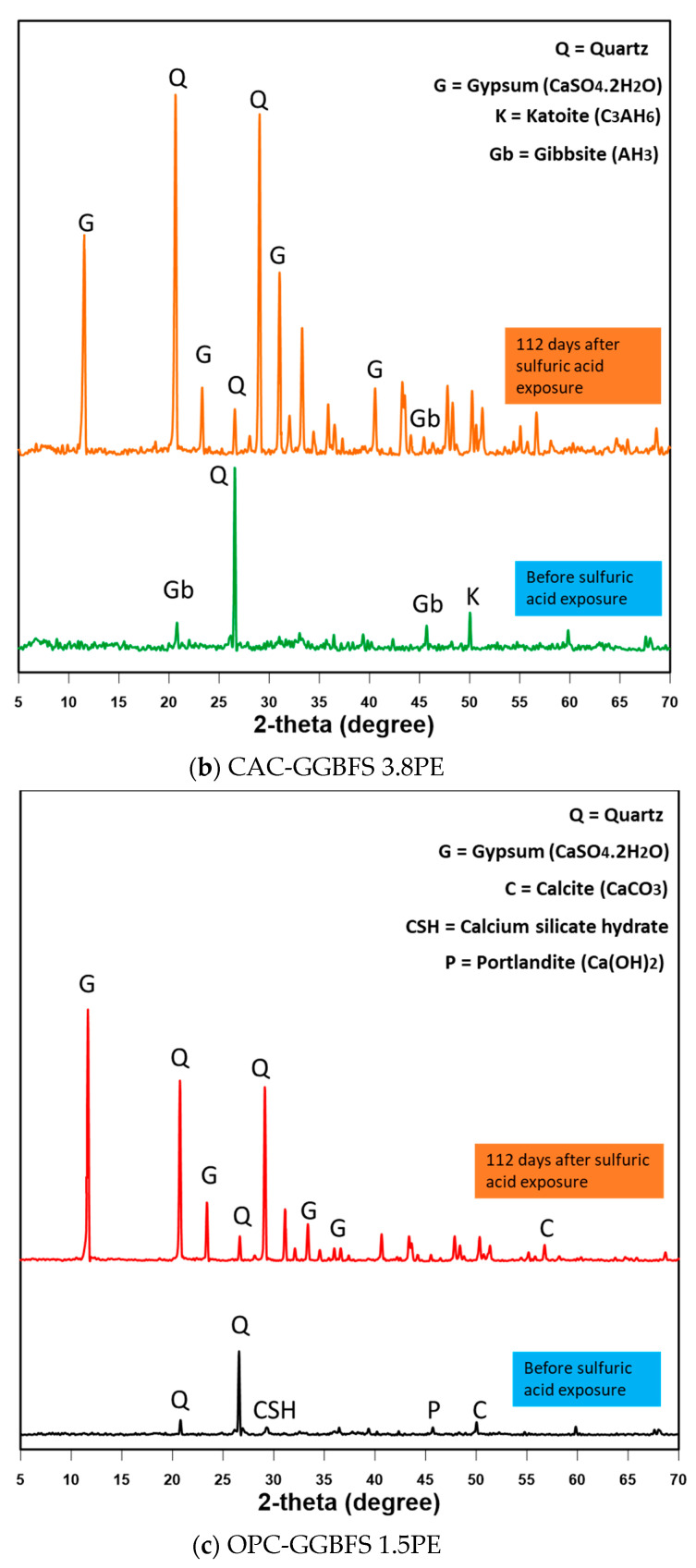
XRD analysis on fibre reinforced CAC/OPC-GGBFS blended mortar.

To identify the corrosion layer product, XRD analysis was conducted on the specimens after 0 and 112 days of sulfuric acid exposure. Figure 8 shows the XRD pattern of powder samples of CAC-GGBFS1.5PE, CAC-GGBFS3.8PE and OPC-GGBFS1.5PE. The initial hydrates produced in the CAC-based binder include katoite (C_3_AH_6_) and gibbsite (AH_3_). Quartz was also observed due to the presence of silica sand (SiO_2_). After 112 days of sulfuric acid attack, the soft deteriorated layers were scraped off the surface of the specimens, dried and powdered for analysis. As shown in Figure 8, the formation of gypsum (CaSO_4_·2H_2_O) was observed within the matrix as a result of sulfur penetration. Katoite is the result of crystallization of gypsum dissociation. The very protruding peak of gypsum is ascribed to the constant diffusion of sulfate ions from the solution. Gibbsite (AH_3_) still existed after 112 days of sulfuric acid exposure, suggesting that AH_3_ is precipitated as an acid-resistant barrier to slow down further corrosion [12]. For OPC-based mortar, initial hydrates of CSH and portlandite (Ca(OH)_2_) were observed as well as quartz. Gypsum (CaSO_4_·2H_2_O) was formed after 112 days sulfuric acid attack and led to the dissolution of CSH and portlandite (Ca(OH)_2_). C-S-H hydrates and portlandite were completely replaced with gypsum in OPC-based samples, whereas gibbsite reappeared (Equations (4) and (5)) in CAC-based samples which can neutralise more acid.

#### 3.2.4. Compressive Strength

**Figure 9:**
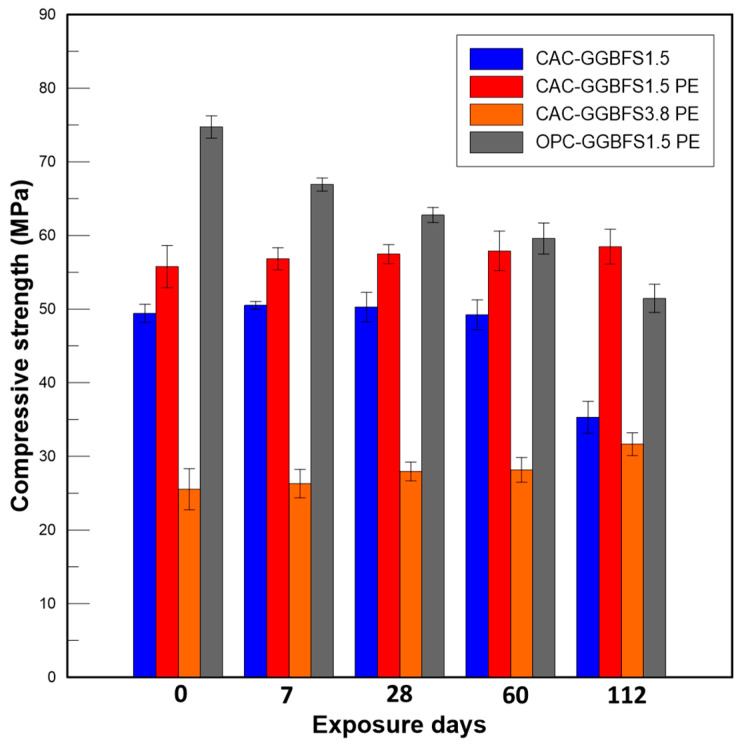
Compressive strength development after sulfuric acid exposure.

The immersed specimens were taken from the sulfuric acid solution after the exposure period of 7, 28, 60 and 112 days and the surfaces were carefully brushed to remove the loose particles. Then, they were left to dry at an ambient temperature for 1 h before testing the compressive strength. Figure 9 shows the compressive strength development of the four mixtures after being immersed in 3% sulfuric acid solution for a period of up to 112 days. The compressive strength of un-exposed CAC-GGBFS1.5, CAC-GGBFS1.5PE, CAC-GGBFS3.8PE and OPC-GGBFS1.5PE at 28 days was 49.42, 59.76, 25.54 and 74.73 MPa, respectively. With the fibre addition, the compressive of CAC-GGBFS1.5PE increased 12%—this can be ascribed to the fibre bridging effect. However, as the s/c ratio increased, the compressive strength of CAC-GGBFS3.8PE at 28 days reduced almost 54%; this was due to the time-consuming pozzolanic activity of minerals. OPC-GGBFS1.5PE showed the highest compressive of 74.73 MPa among the four mixtures before acid exposure. After sulfuric acid attack, the compressive strength of CAC-based samples was slightly increased, while that of the OPC-based sample was sharply reduced from 74.73 to 66.91 MPa after only 7 days of sulfuric acid exposure. The strength gain of CAC-based samples with PE fibres is associated with the growth of ettringite in pores, which could fill the pores and therefore densify the interface between the fibre and the matrix, resulting in higher fibre bridging strength. The compressive strength of OPC-GGBFS1.5PE reduced continuously until the end of exposure for 112 days to 51.45 MPa, a 31% reduction in its original strength, with a large amount of soft expansive gypsum layer in the cementitious matrix, therefore loosening the fibre-matrix bonding strength. For the CAC-GGBFS1.5 group after acid exposure, a rapid reduction of 28% was observed from 49.23 (60 days) to 35.29 MPa (112 days); similar results were also found in [26,27,28]. This is due to the cement hydrates reacted with sulfate ions (SO_4_^2−^) until the pressure exceeded the tensile strength of mortar, then cracks formed and resulted in surface cracking and spalling. However, for CAC-GGBFS1.5PE and CAC-GGBFS3.8PE groups, neither the compressive strength reduction was found in Figure 9 nor surface cracking was observed in Table 3. A similar trend was observed for these two groups, where the compressive strength increased 4% and 5%, respectively, from unexposed to 112 days of acid exposure. This could be attributed to the fact that PE fibre-reinforced mortar has a much higher tensile performance with tiny cracks. The tiny crack width can keep the sulfate ion (SO_4_^2−^) diffusion low and prevent accelerated corrosion. Additionally, even when fibre-reinforced samples finally cracked, the crack width was much smaller than plain mortar samples, thereby slowing down the dramatic increase in the aggressive ion diffusion into the cement matrix and preventing the deterioration rate from speeding up. Therefore, PE fibre reinforced CAC-GGBFS mortar can provide a better acid resistance than plain mortar and OPC-based specimens. The excellent behaviour of the CAC-based specimen is highly desirable for sewerage pipes in sulfuric acid rich environments.

#### 3.2.5. Tensile Stress-Strain Relationship

**Figure 10:**
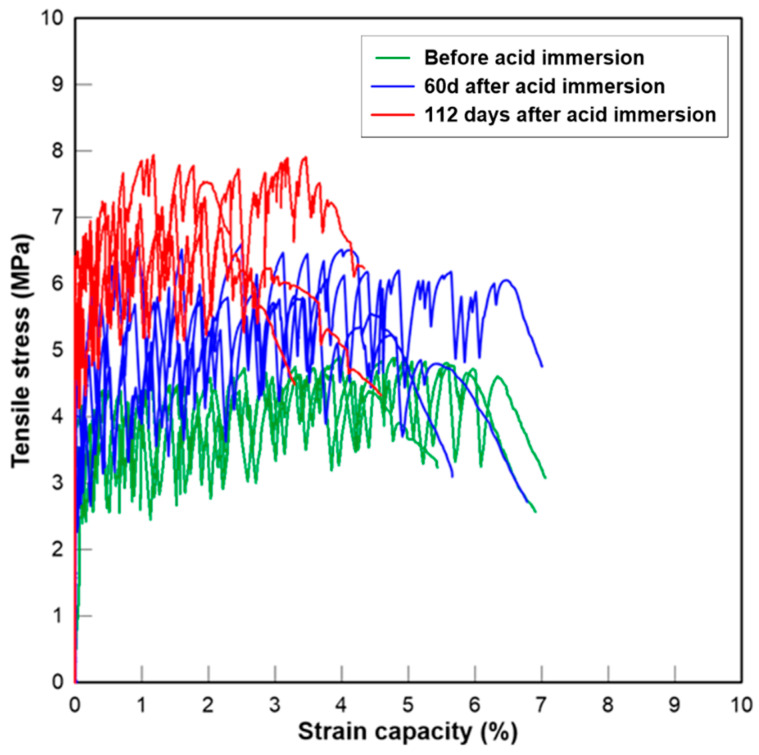
Tensile stress-strain curves of CAC-GGBFS 1.5PE.

**Figure 11:**
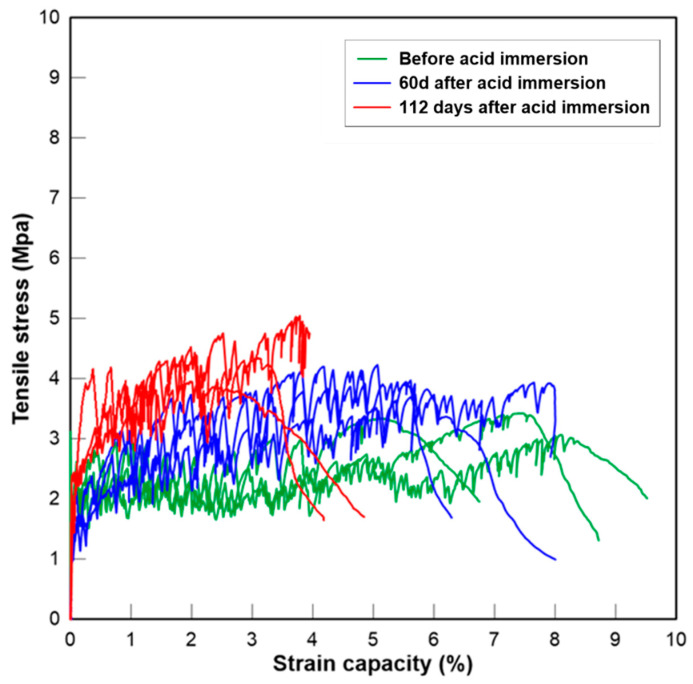
Tensile stress-strain curves of CAC-GGBFS 3.8PE.

**Figure 12:**
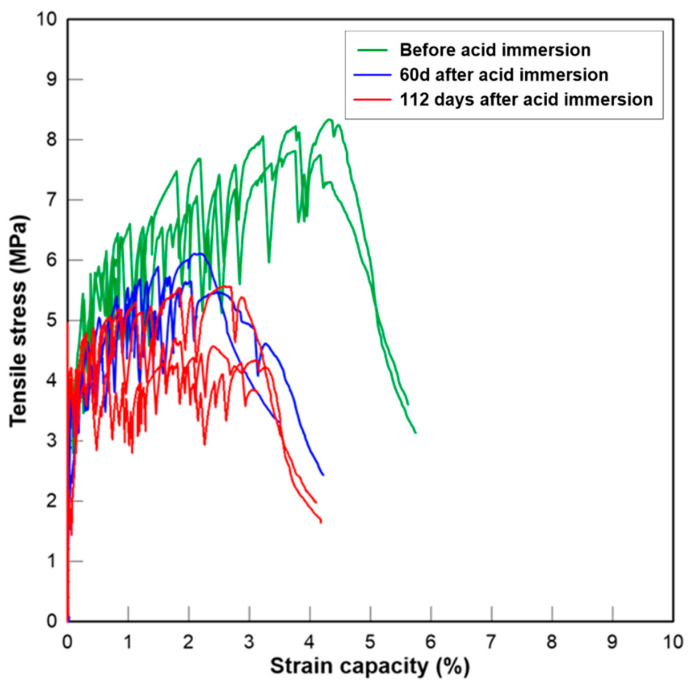
Tensile stress–strain curves of OPC-GGBFS 1.5PE.

The curves of the tensile stress–strain relationship of three groups of PE fibre reinforced mortar are presented below at the age of 0, 60 and 112 days after initial acid exposure. The critical parameters representing the tensile properties of fibre-reinforced mortar before and after acid exposure in this study, which include the initial cracking strength (*σ_tc_*), the peak stress (*σ_tu_*), the strain capacity corresponding to the peak stress (*ε_tu_*), as well as crack numbers (*N_c_*) and crack width (*w_c_*), are tabulated in Table 5. Overall, the change in tensile strength agreed with the changes in compressive strength after sulfuric acid exposure. The tensile strength of two CAC-based mixtures exposed to H_2_SO_4_ solution increased over time. The strength development is again due to the continuous hydration of cement, the pozzolanic reaction of GGBFS and the reaction between SO_4_^2−^ and hydration products. The interface between the fibres and the matrix could be densified after the reactions, resulting in higher fibre bridging strength. The tensile stress–strain curves of CAC-GGBFS1.5PE are shown in Figure 10. Before acid exposure, the initial cracking and ultimate cracking strength of CAC-GGBFS1.5PE was 4.05 and 5.46 MPa, respectively. The tensile strain capacity reached almost 5.78%. The strain capacity was not affected after 60 days of sulfuric acid exposure, but the tensile strength increased 12%. After 112 days of sulfuric acid exposure, the ultimate tensile strength increased 26% but the strain capacity reduced by almost 50%. Figure 11 presents the results of CAC-GGBFS3.8PE. Increased s/c ratio led to a reduction in initial cracking and ultimate tensile strength by 46% and 43%, respectively. This was attributed to the lower cement content after GGBFS substitution that slowed down the pozzolanic reaction. Nevertheless, the increased amount of GGBFS enhanced the tensile strain capacity from 5.78% up to 7.42%. On exposure to sulfuric acid, it is clearly observed that both the initial cracking strength and the ultimate cracking strength of CAC-GGBFS3.8PE increased. A main reason for the strength growth of CAC-GGBFS1.5PE and CAC-GGBFS3.8PE could be due to the alumina hydrate and less calcium hydroxide in CAC-based mortar, which slowed down the corrosion process and the core of the specimen maintained its structural integrity. On the contrary, due to the thick corrosion layer, 5.09 mm from each side, as shown in Table 4, the OPC-based samples experienced a reduction in the tensile parameters. Gypsum has occupied almost 80% of the sample cross section. The tensile behaviour of OPC-GGBFS1.5PE is shown in Figure 12. After a long-term attack by sulfuric acid solution, the free H^+^ and sulfate ions weakened its matrix, thereby leading to reductions of 26% in the first cracking strength and 34% in the ultimate tensile strength after 112 days of acid exposure.

#### 3.2.6. Morphologies of the Pull-Out PE Fibre

**Figure 13:**
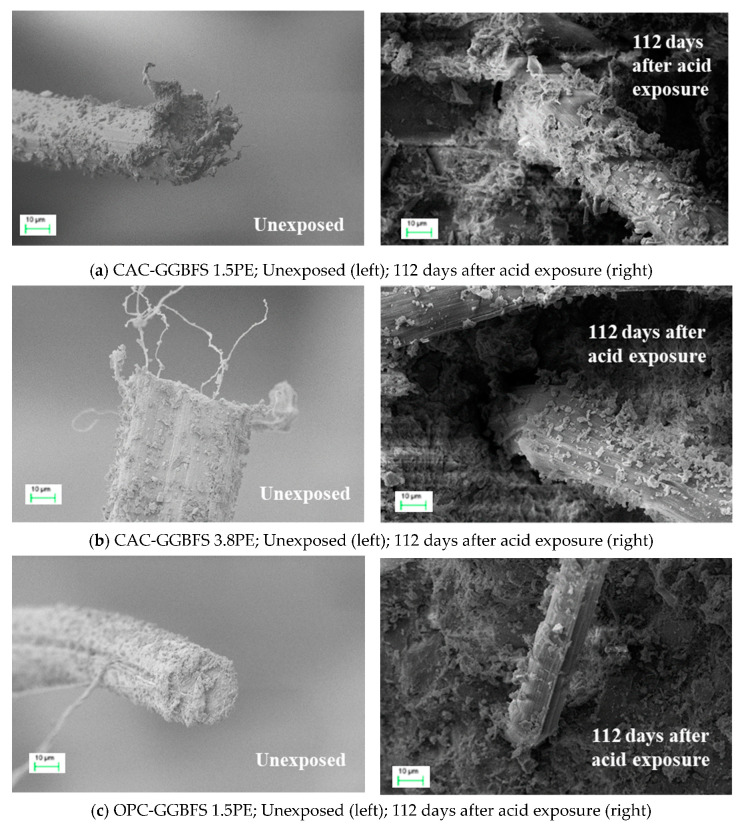
SEM images of PE fibre surface morphology before and 112 days after acid exposure [17]; (**a**) PE fibre surface morphology (CAC-GGBFS 1.5PE); (**b**) PE fibre surface morphology (CAC-GGBFS 3.8PE); (**c**) PE fibre surface morphology (OPC-GGBFS 1.5PE).

Figure 13a–c show SEM images of the fracture surface of PE fibre after pull-out from matrices. All samples showed that the PE fibre surface was grooved severely before and after 112 days of acid exposure during the pull-out process, means that the fibre/matrix interface remained stable after acid attack. Compared with CAC-GGBFS 3.8PE in Figure 13b and OPC-GGBFS 1.5PE in Figure 13c, CAC-GGBFS 1.5PE shows that the fibre surface was stuck to more matrix particles during the pull-out process, as shown in Figure 13a. The substantial residual mortar content contributes to the resistance to fibre pull-out load, leading to a higher tensile stress. Furthermore, it is worth noting that, due to the semi-crystalline and no-polar-group structure, the PE fibres show an excellent chemical resistance. Thus, it is likely that no deterioration in the mechanical performance of PE fibres under the aggressive agents occurred, as observed in SEM images.

#### 3.2.7. Crack Characteristics

Table 5 lists the crack information of CAC-GGBFS1.5PE, CAC-GGBFS3.8PE and OPC-GGBFS1.5PE at the age of 0, 60 and 112 days initial after sulfuric acid exposure. After sulfuric acid attack, PE fibre-reinforced specimens still exhibited significant multiple cracking behaviours, which resulted in a low hydraulic conductivity even in the cracked stage. The cracking behaviour before and after sulfuric acid exposure can reflect the tensile properties of fibre-reinforced mortar to a certain extent. The characteristics of the cracks after failure, e.g., the number of cracks (*N_c_*) and the average crack width (*w_c_*), are determined by the crack pattern. The number of cracks (*N_c_*) of the specimen was visually obtained and we counted the cracks on both sides of specimen. Most of the micro-cracks were found that went through entire section of the specimen. The average crack width (*w_c_*) was then calculated as:

(8) $$\mathrm{Average}\;\mathrm{crack}\;\mathrm{width}=\frac{Tensile\;strain\times gauge\;length\left(80\;mm\right)}{Average\;crack\;number}$$

From Table 6, two CAC-based mixture groups showed a positive change in crack width. The average crack width of CAC-GGBFS1.5PE reduced from 182 (unexposed) to 137 μm (112 days exposed). The crack width also decreased with the increased amount of GGBFS, as can be seen in CAC-GGBFS3.8PE, due to its finer particle size and irregular morphology. It is clear that the multiple cracking behaviour of PE fibre-reinforced CAC-GGBFS mortar was promoted by the increased s/c ratio. The decrease in the average crack width is helpful for limiting the diffusion of free H^+^ and sulfate ions into the interior of the pipes, although it results in slightly reducing the tensile ductility. It can slow the deterioration and enhance the durability of sewerage infrastructures. OPC-GGBFS1.5PE showed less change compared with the other two groups, from 159 to 151 μm. The change in crack width is also associated with the fibre-matrix interfacial properties change, as discussed in Section 3.2.6.

#### 3.2.8. Retention Coefficient

**Figure 14:**
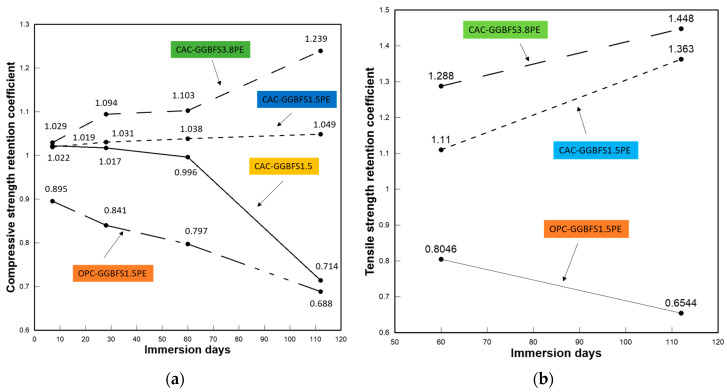
Sulfuric acid resistance of all mixtures. (**a**) Compressive stress. (**b**) Ultimate tensile stress.

The residual mechanical properties of all mixtures after sulfuric acid attack was characterized by the retention coefficient (*R*), which is calculated by using the residual tensile or compressive strength of the specimen after acid immersion (*Sx*) divided by the ultimate tensile or compressive strength of the specimen before acid immersion (*Sy*), *R* = *Sx*/*Sy*. Figure 14 presents the retention coefficients corresponding to the ultimate tensile stress and compressive strength obtained in the uniaxial tension and compression tests, respectively. The compressive strength retention coefficients of CAC-GGBFS1.5, CAC-GGBFS1.5PE, CAC-GGBFS3.8PE and OPC-GGBFS1.5PE were 0.714, 1.049, 1.239 and 0.688, respectively, after 112 days of acid exposure. CAC-GGBFS1.5 showed a gradual decrease from unexposed to 60 days of exposure to sulfuric acid due to the generation of gypsum causing internal pressure, leading to surface spalling. Compared with CAC-GGBFS1.5, the CAC-based mixtures with PE fibres were observed to have an ascending trend. OPC-GGBFS1.5PE’s trend descended continuously from 7 days of acid exposure until the end of 112 days. The ultimate tensile strength retention coefficients of CAC-GGBFS1.5PE, CAC-GGBFS3.8PE and OPC-GGBFS1.5PE were 1.363, 1.448 and 0.654, respectively. As with the compressive behaviour, the coefficients of CAC-based mixtures were increased but those of the OPC-based mixture were decreased. Additionally, GGBFS was proved to improve acid resistance, as the compressive and tensile retention coefficients of CAC-GGBFS3.8PE were 15% and 5% higher than CAC-GGBFS1.5PE. Therefore, based on the available data, the use of a CAC-based binder with PE fibre shows superior performance, not only in the mechanical behaviour but also in the durability after acid resistance.

## 4. Conclusions

The performance of CAC-GGBFS blended mortar, PE fibre-reinforced CAC-GGBFS blended mortar and PE fibre-reinforced OPC-GGBFS blended mortar was assessed in the aggressive testing conditions of 3% sulfuric acid for up to 112 days. Based on visual assessment, physical, mechanical and microstructure analysis, the following conclusions can be drawn:

1. Under normal environmental conditions, OPC-based binder system has a better performance to water absorption and sorptivity. The addition of PE fibre leads to a decrease in the specimen’s porosity.
2. PE fibre-reinforced CAC-GGBFS blended mortar performed significantly better than plain CAC-GGBFS blended mortar and the OPC-based mixture in sulfuric acid solution in terms of visible deterioration and change of cross-section.
3. The main deterioration mechanism in the sulfuric acid testing indicated from XRD analysis was found to be the formation of gypsum on the exterior surfaces of the specimen for the sample without fibre reinforcement, followed by surface delamination and spalling. The main crystalline products in the samples after sulfuric acid attack are gypsum in both CAC-based and OPC-based mixtures. The reappearance of gibbsite in CAC-based samples can neutralise more acid and result in a better performance than the OPC-based system.
4. Long-term exposure to aggressive solutions leads to the increase in compressive strength and tensile strength but a reduction in tensile strain capacity of CAC based samples. Nevertheless, it still exhibits strain-hardening behaviour by showing multiple cracks with desirable high tensile ductility.

The durability performance of concrete sewerage pipes is a significant concern for local water utility as the demand for urban infrastructures to have a long service life and minimal maintenance costs is needed. One of the major causes of concrete sewerage pipe deterioration globally is sulfuric acid (H_2_SO_4_) attack from acid generated from sewerage systems. The growing demand for more cost-effective repair options has made PE fibre-reinforced CAC-GGBFS blended mortar an alternative for the wastewater industry. This research highlights that PE fibre-reinforced CAC-GGBFS blended mortar provides improved durability to sulfuric acid attack, which can add longevity and decrease the maintenance costs of concrete sewerage pipes.

## Figures and Tables

**Table 1:** Properties of polyethylene (PE) fibre.

PE Fibre Properties	Diameter (µm)	Fibre Length (mm)	Fibre Aspect Ratio	Nominal Strength (MPa)	Young’s Modulus in Tension (GPa)	Elongation at Break (%)	Specific Gravity
Value	20	18	900	3000	100	3	0.97

**Table 2:** Mix proportions of plain and PE fibre-reinforced mortar (kg/m^3^) [17].

Mixture ID	Cement	GGBFS	Water	Fine Silica Sand	HPMC ^#^	PCE *	PE Fibre (1% vol)	w/b ^^^	s/c ^+^
CAC-GGBFS 1.5 *(Control)*	480	720	370	594	---	3.7	---	0.3	1.5
CAC-GGBFS 1.5PE	480	720	370	594	0.5	2.2	10	0.3	1.5
CAC-GGBFS 3.8PE	240	954	370	594	0.5	2.5	10	0.3	3.8
OPC-GGBFS 1.5PE *(referenced OPC mixture)*	480	720	370	594	0.5	1.14	10	0.3	1.5

^#^ Hydroxypropyl methylcellulose; * powder type superplasticiser (Polycarboxylates); ^^^ water to binder ratio; ^+^ slag to cement ratio.

**Table 3:** Visual observation of samples after different age of acid exposure.

Exposure Days	CAC-GGBFS 1.5 *(Control)*	CAC-GGBFS 1.5PE	CAC-GGBFS 3.8PE	OPC-GGBFS 1.5PE *(OPC Reference)*
0 (Unexposed)	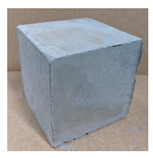	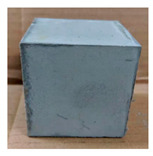	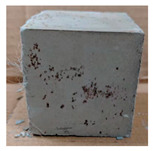	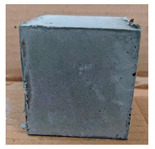
7	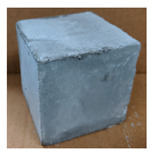	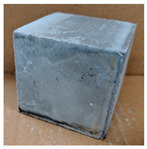	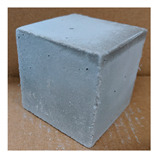	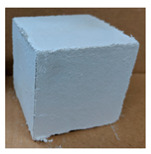
28	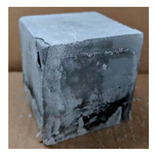	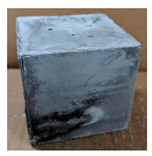	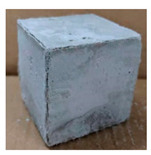	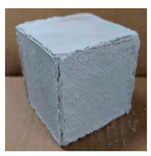
60	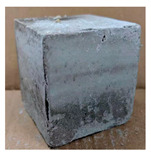	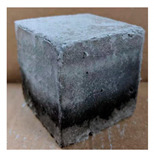	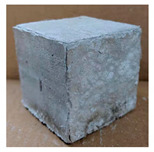	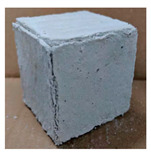
112	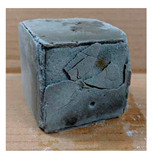	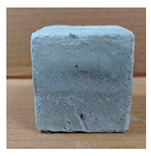	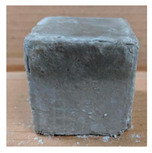	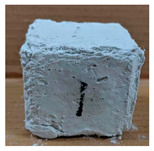

**Table 4:** Change of cross-section at different age after acid exposure.

Exposure Days	CAC-GGBFS 1.5 *(Control)*	CAC-GGBFS 1.5PE	CAC-GGBFS 3.8PE	OPC-GGBFS 1.5PE *(OPC Reference)*
0 (Unexposed)	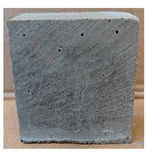	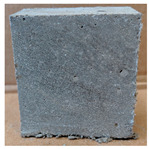	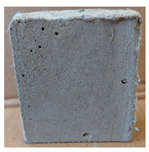	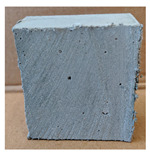
7	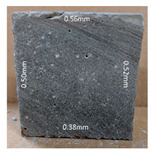 Average layer thickness: 0.49 mm	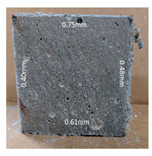 Average layer thickness: 0.56 mm	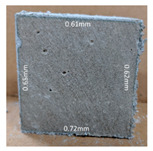 Average layer thickness: 0.65 mm	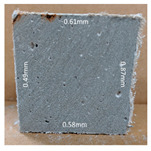 Average layer thickness: 0.64 mm
28	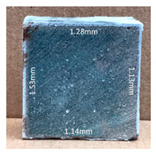 Average layer thickness: 1.27 mm	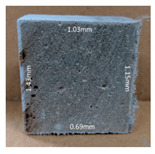 Average layer thickness: 1.07 mm	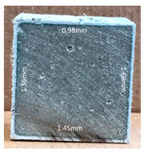 Average layer thickness: 1.37 mm	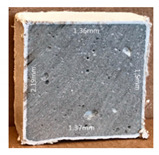 Average layer thickness: 1.61 mm
60	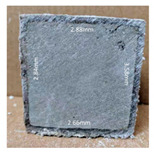 Average layer thickness: 2.99 mm	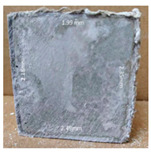 Average layer thickness: 2.07 mm	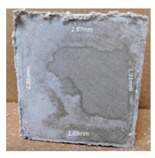 Average layer thickness: 2.28 mm	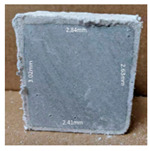 Average layer thickness: 2.72 mm
112	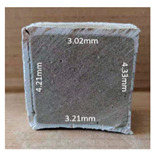 Average layer thickness: 3.69 mm	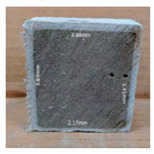 Average layer thickness: 3.26 mm	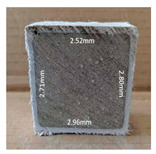 Average layer thickness: 2.75 mm	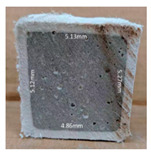 Average layer thickness: 5.09 mm

**Table 5:** Tensile properties of fibre reinforced mortar before and after sulfuric acid exposure.

Exposure Days	Mixture ID	Initial Cracking Strength *σ_tc_* (MPa)	Ultimate Tensile Strength *σ_tu_* (MPa)	Tensile Strain Capacity *ε_tu_* (%)
0 day	CAC-GGBFS 1.5PE	4.05 ± 0.93	5.46 ± 1.14	5.78 ± 0.58
	CAC-GGBFS 3.8PE	2.18 ± 0.23	3.06 ± 0.28	7.42 ± 2.27
	OPC-GGBFS 1.5PE	5.51 ± 0.87	7.32 ± 1.12	4.59 ± 1.46
60 days	CAC-GGBFS 1.5PE	4.59 ± 0.49	6.06 ± 0.26	5.41 ± 0.93
	CAC-GGBFS 3.8PE	2.41 ± 0.45	3.94 ± 0.24	6.91 ± 1.20
	OPC-GGBFS 1.5PE	4.04 ± 0.36	5.89 ± 0.38	2.84 ± 0.58
112 days	CAC-GGBFS 1.5PE	6.01 ± 0.28	7.44 ± 0.48	2.93 ± 0.45
	CAC-GGBFS 3.8PE	2.58 ± 0.10	4.43 ± 0.42	3.31 ± 0.33
	OPC-GGBFS 1.5PE	4.03 ± 0.13	4.79 ± 0.51	2.55 ± 0.30

**Table 6:** Crack information of fibre reinforced mortar before and after sulfuric acid exposure.

Exposure Days	Mixture No.	Crack Numbers *N_c_*	Crack Width *w_c_* (µm)
0 day	CAC-GGBFS 1.5PE	21 ± 0.81	182 ± 0.95
	CAC-GGBFS 3.8PE	32 ± 2.68	135 ± 0.50
	OPC-GGBFS 1.5PE	18 ± 1.70	159 ± 0.55
60 days	CAC-GGBFS 1.5PE	19 ± 1.65	177 ± 0.78
	CAC-GGBFS 3.8PE	27 ± 2.42	127 ± 0.33
	OPC-GGBFS 1.5PE	15 ± 1.45	155 ± 0.46
112 days	CAC-GGBFS 1.5PE	17 ± 1.33	137 ± 0.84
	CAC-GGBFS 3.8PE	23 ± 2.82	115 ± 0.13
	OPC-GGBFS 1.5PE	14 ± 1.02	151 ± 0.14
